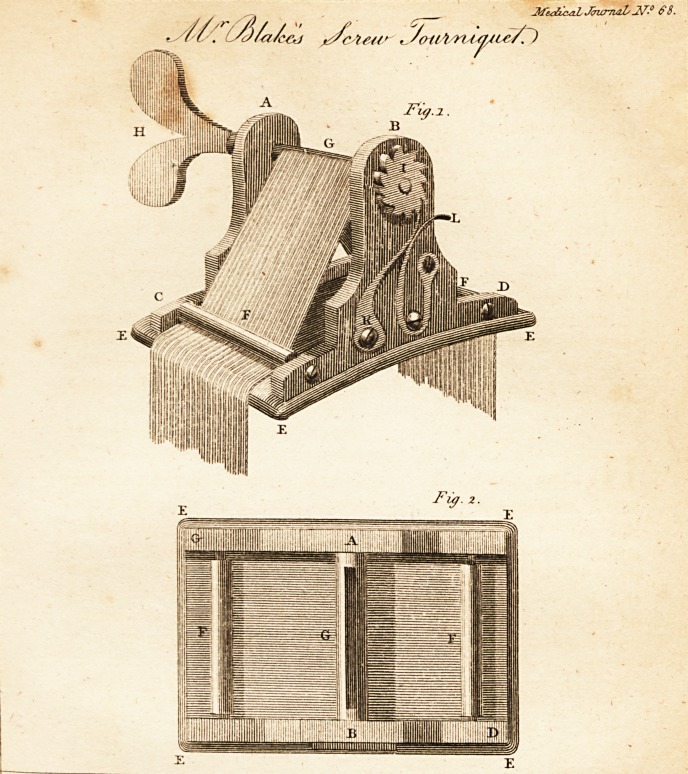# Mr. Blake's Improved Tourniquet

**Published:** 1804-10-01

**Authors:** Andrew Blake

**Affiliations:** Dublin


### *To the Editors of the Medical and Physical Journal.*

Gentlemen,

HAVING observed some inconveniencies in the received screw tourniquet, arising from its size and weight, I was led to consider how they might be obviated; and after some attention to the subject, have constructed one, a drawing of which I send you, which will, I hope, have the desired effect. It likewise appears worthy the attention of the army, as it is considerably cheaper than the screw, and much more convenient than the field tourniquet.

*Dublin,* I am, &c.

*July* 11, 1804. ANDREW BLAKE.

---

### EXPLANATION OF THE PLATE.

Fig. 1. Is a perspective view of the instrument.

2. Is a geometrical view of the lower part of it, shewing the situation of the pieces FF and C.

ABCD Is the brass frame of the apparatus, of which A and B are the upright pieces, and CD the bottom plate.

EEE Is the pad.

FF Are rollers under which the strap passes.

G Is a similar roller of a large size, having a slit through it of sufficient dimensions to admit the strap.

H Is a flier, by which the roller C may be turned, and consequently a portion of the strap wound up on it.

I Is a rotchet wheel fixed to its other end, and which with the springs K and L, prevent the roller from running back; by pressing on the end of the spring L, the wheel I, together with the roller G, and the strap, is released.

The letters of reference to Fig. 2, are affixed respectively to the same parts of the apparatus as in Fig. 1.

Case

*Medical Journal No. 68.*

## Figures and Tables

**Fig.1. Fig. 2. f1:**